# Gut microbiota reconstruction after liver transplantation and its association with early postoperative infections in patients with liver failure

**DOI:** 10.3389/fcimb.2026.1845273

**Published:** 2026-06-08

**Authors:** Bing Chen, Jun Chen, Zhangpeng Feng, Hanbei Lv, Qinghong Lin, Guoping Jiang

**Affiliations:** 1 Key Laboratory of Artificial Organs and Computational Medicine of Zhejiang Province, Shulan (Hangzhou) Hospital, Shulan International Medical College, Zhejiang Shuren University, Hangzhou, China; 2 Department of General Surgery, The Second People’s Hospital of Guizhou Province, Guiyang, Guizhou, China; 3 School of Medicine, Zhejiang Chinese Medical University, Hangzhou, Zhejiang, China

**Keywords:** gut microbiota, liver failure, liver transplantation, postoperative infection, probiotics

## Abstract

**Background:**

Postoperative infection remains a major cause of morbidity after liver transplantation (LT) in patients with liver failure. Increasing evidence suggests that gut microbiota dysbiosis may contribute to infection risk, but its dynamic changes after LT are not fully understood.

**Methods:**

This retrospective study included 60 patients with liver failure who underwent LT and developed postoperative infection-related risk. Patients were divided into a probiotic group and a non-probiotic group. Fecal samples were collected before transplantation and on postoperative days 7, 14, 21, and 28. Metagenomic sequencing was performed to analyze gut microbial composition, diversity, and antibiotic resistance genes.

**Results:**

The probiotic group showed a significantly lower rate of postoperative bacterial infection, especially intra-abdominal infection. After LT, gut microbiota gradually recovered in both groups, but restoration was faster in the probiotic group. The non-probiotic group showed persistent dysbiosis, characterized by enrichment of opportunistic pathogens such as *Enterococcus* and *Klebsiella*, whereas beneficial genera including *Bifidobacterium* and *Lactobacillus* were more abundant in the probiotic group. Antibiotic resistance genes were also more enriched in the non-probiotic group.

**Conclusion:**

Early postoperative gut microbiota reconstruction is closely associated with infectious complications after LT, and modulation of gut microbiota may help improve postoperative outcomes.

## Introduction

Liver failure is a severe clinical syndrome caused by various etiological factors that lead to profound impairment of hepatic functions, including synthesis, detoxification, and biotransformation (Bajaj et al., 2022; Br and Sarin, 2023). It is clinically characterized by jaundice, coagulopathy, and hepatic encephalopathy, and often progresses rapidly with poor prognosis and high mortality due to the combined effects of immune-mediated injury, ischemia–hypoxia, and endotoxemia (Br and Sarin, 2023; Artru and McPhail, 2024). The etiological profile varies geographically, with alcohol consumption and drug-induced liver injury predominating in Western countries, whereas hepatitis B virus (HBV) infection remains the leading cause in China (Bhatti et al., 2023; Kezer et al., 2023). Currently, liver transplantation (LT) is the most effective treatment for liver failure and significantly improves patient survival (Kulkarni et al., 2024). According to data from the China Liver Transplant Registry, the 1-year postoperative survival rate reaches approximately 84.7%. However, long-term outcomes remain limited by postoperative complications, including graft rejection, infections, and biliary complications. Among these, infection is particularly common, with an incidence of 53%–79% (Madreseh et al., 2020; Hernaez et al., 2023). Patients with liver failure often present with critical illness and poor preoperative conditions, and surgical trauma together with the use of high-dose corticosteroids and immunosuppressive agents further increases the risk of infection, contributing to a perioperative mortality rate of 14%–25% (Chesdachai et al., 2022; Lin et al., 2018; Molinari et al., 2019). Effective strategies for preventing and managing post-transplant infections remain limited, making this a major challenge in organ transplantation.

The liver and intestine are anatomically and functionally interconnected through the portal vein, lymphatic system, and enterohepatic circulation, forming a close liver–gut axis (Smith et al., 2024). Microbial metabolites and toxins derived from the intestine can enter the liver via the portal circulation, while the liver regulates intestinal immunity and microbial homeostasis through the secretion of bile acids and other bioactive molecules (Brandl et al., 2017; Tripathi et al., 2018). The gut microbiota is considered an important metabolic “organ” of the human body and is commonly classified taxonomically at different levels. At the phylum level, it is mainly composed of Firmicutes and Bacteroidetes, along with Actinobacteria and Proteobacteria (Lloyd-Price et al., 2016; Rinninella et al., 2019). These microbial communities play essential roles in host health, including food digestion and absorption, vitamin synthesis, immune regulation, metabolic homeostasis, and resistance to pathogenic colonization (Rowland et al., 2018). Post–LT infection is closely associated with the recipient’s immune status. Through the gut–liver axis and enterohepatic circulation, the gut microbiota plays a critical role in hepatic immunity, inflammation, and liver injury, and its importance in transplantation has gained increasing attention (Tripathi et al., 2018; Sucu et al., 2023). Therefore, exploring the relationship between LT for liver failure and the gut microbiota, as well as investigating microbiota-targeted interventions to reduce post-transplant infections and improve clinical outcomes, may have important clinical significance.

Although accumulating evidence suggests that the gut microbiota is closely associated with post–LT infections, the underlying mechanisms remain incompletely understood. In this study, we retrospectively collected gut microbiota data from patients with liver failure who developed infections after LT and systematically analyzed the characteristics of their microbial community structure. The aim was to explore the association between alterations in gut microbiota and postoperative infections, thereby providing potential evidence for early prediction and clinical prevention of post-transplant infections.

## Materials and methods

### Patient selection and study design

The present retrospective study included 60 patients who underwent modified piggyback liver transplantation for liver failure secondary to hepatitis B virus infection, alcohol-related liver disease, or drug-induced liver injury and subsequently developed postoperative infections at our center between January 2022 and December 2023. In the present study, postoperative infections after LT were defined as respiratory tract, intra-abdominal, biliary tract, wound, urinary tract, and bloodstream infections; the diagnostic criteria are summarized in **Supplementary Table 1**. All surgeries were performed by the same surgical team. The study was approved by the hospital’s ethics committee, and all participants provided written informed consent.

### Inclusion and exclusion criteria

Patients with postoperative infections after LT were included in the present study. The inclusion criteria were as follows: (1) diagnosis of liver failure and receipt of LT; (2) age 18–70 years; (3) no severe heart, kidney, or lung failure; (4) no infection within 1 week before surgery; and (5) written informed consent from the patients and their families. The exclusion criteria were as follows: (1) malignant tumors; (2) relaparotomy within 1 month after surgery for any reason, or postoperative bile, intestinal, or pancreatic leakage; (3) retransplantation; (4) suspected or confirmed postoperative immune rejection treated with steroid pulse therapy; (5) preoperative intestinal obstruction or perforation; (6) perioperative oral antibiotics were administered, and (7) incomplete clinical data.

### Perioperative immunosuppressive therapy and nutritional protocols

During LT, patients received prophylactic antibiotics, basiliximab induction therapy, and methylprednisolone pulse therapy. After transplantation, a triple immunosuppressive regimen consisting of tacrolimus, methylprednisolone, and mycophenolate mofetil/sodium was administered. All patients were managed according to a standardized institutional perioperative nutritional protocol for liver transplantation. Preoperatively, nutritional status was assessed by the transplant and clinical nutrition teams, and individualized nutritional support was provided based on clinical condition. Postoperatively, nutritional support was jointly managed by the transplant, intensive care, and nutrition teams. Enteral nutrition was preferred when feasible, with parenteral nutrition used as temporary or supplemental support if needed. Nutritional targets were approximately 25–30 kcal/kg/day and 1.2–1.5 g/kg/day protein, adjusted according to clinical status. Feeding was gradually advanced as tolerated.

### Sample and data collection

Fecal samples were collected from each liver transplantation recipient at five time points: 1 day before transplantation and 7, 14, 21, and 28 days after transplantation. At each time point, at least 0.6 g of fecal material was obtained under strict aseptic conditions to minimize microbial contamination. All samples were stored at −80 °C until DNA extraction. Clinical data and laboratory parameters were extracted from the electronic medical record system. Laboratory values were collected during the early postoperative period and matched as closely as possible to the fecal sampling schedule, including preoperative baseline and postoperative days 7, 14, 21, and 28. When multiple results were available on the same day, the value closest to fecal sample collection was used for analysis. These parameters were analyzed to support the assessment of postoperative inflammatory status and infectious complications.

### Oral probiotics

To further clarify the potential impact of probiotic supplementation on bacterial infections following LT, all included patients were categorized into either the probiotic group or the non-probiotic group. Among patients assigned to the probiotic group, probiotic treatment was commenced 6 hours after LT and administered either orally or via a nasogastric tube. The probiotic product used in this study was Weileshu [Jinqi Biotechnology (Anhui) Co., Ltd.]. Each sachet contained 20 billion CFU (2.0 × 10¹^0^ CFU) of live probiotics, mainly comprising *Lactobacillus helveticus* R52, *Ligilactobacillus salivarius* LI01, and *Bifidobacterium longum* subspecies. The dosage was 2 g twice daily. In patients receiving antibiotics, probiotic supplementation was administered 2 hours after antibiotic use and was maintained through postoperative day 14. For administration, one 2 g sachet of Weileshu was freshly dissolved in water at room temperature or lukewarm temperature and administered orally or through a nasogastric tube, with the tube flushed with water before and after delivery to ensure complete administration and prevent tube obstruction.

### DNA library preparation and illumina sequencing

For each sample, 1 μg of genomic DNA was randomly fragmented into ~350-bp fragments using a Covaris ultrasonicator (Gene Company Limited, China) for library construction. Libraries were quantified with Qubit 2.0, assessed using the Agilent 2100 Bioanalyzer, and accurately quantified by Q-PCR; libraries with an effective concentration >3 nM were pooled and subjected to Illumina PE150 sequencing.

### Metagenomic assembly, gene prediction, and functional annotation

Raw data were preprocessed using Readfq to generate clean data, which were assembled with MEGAHIT to obtain scaftigs. ORFs in scaftigs ≥500 bp were predicted using MetaGeneMark, and the resulting unigenes were used for downstream analyses. Based on gene abundance, basic statistics, core-pan gene analysis, inter-sample correlation analysis, and Venn diagram analysis were performed. Unigenes were aligned to the KEGG database using DIAMOND for taxonomic and functional annotation, and to the CARD database for antimicrobial resistance gene annotation.

### Statistical analysis

All metagenomic analyses were conducted in R. Alpha diversity was assessed using the ACE, Chao1, OBS, Shannon, and Simpson indices, whereas beta diversity was evaluated by PCA and PCoA based on Bray–Curtis distances. LEfSe analysis was performed to identify taxa with significantly different relative abundances between groups (LDA score >2.0, P < 0.05). PICRUSt was used to predict microbial functions based on the KEGG database. Group differences were analyzed using the Wilcoxon rank-sum test, and temporal changes in the gut microbiota within 1 month after LT were assessed using the Kruskal–Wallis test. A P value <0.05 was considered statistically significant.

Clinical data were analyzed using SPSS version 26.0. Non-normally distributed variables are presented as the median and interquartile range [M (IQR)] and were compared using the Mann–Whitney U test. Categorical variables are expressed as number and percentage [n (%)] and were compared using the chi-square test. A two-sided P < 0.05 was considered statistically significant, with a significance level of α = 0.05.

## Result

### Clinical characteristics

Between January 2022 and December 2023, 60 patients who underwent liver transplantation for liver failure at Shulan (Hangzhou) Hospital were retrospectively enrolled in the present study. To better characterize the influence of probiotic intervention on postoperative bacterial infection across different liver failure subtypes, patients were categorized into three equally sized groups: acute liver failure (n = 20), acute-on-chronic liver failure (n = 20), and chronic liver failure (n = 20). The study cohort was further divided into a probiotic group (n = 30) and a non-probiotic group (n = 30). Baseline demographic and clinical data are presented in **Table 1**.

**Table 1:** Clinical characteristics of 60 patients undergoing liver transplantation for liver failure.

Clinical variables	probiotic group (n=30)	Non-probiotic group (n=30)	Z/χ2	P-value
Sex			0.082	0.774
Male	22(73.3%)	21(70.0%)		
Female	8(26.7%)	9(30.0%)		
Age (years)	51.0(48.0-60.0)	48.0(43.0-51.0)	1.532	0.126
BMI	22.2(20.0-25.1)	21.2(20.3-23.2)	0.562	0.574
Etiology			0.001	1.000
Acute liver failure	10(33.3%)	10(33.3%)		
Acute-on-chronic liver failure	10(33.3%)	10(33.3%)		
Chronic liver failure	10(33.3%)	10(33.3%)		
Type of transplantation			0.001	1.000
Whole liver	27(90.0%)	27(90.0%)		
Split liver	3(10.0%)	3(10.0%)		
ABO compatibility			0.162	0.588
Yes	26(86.7%)	27(90.0%)		
No	4(13.3%)	3(10.0%)		
MELD score	33.0(28.0-40.0)	34.0(26.0-40.0)	0.269	0.788
Operation time (min)	414.0(288.0-517.0)	360.5(311.5-390.0)	1.281	0.200
Transfusion volume (U)	9.0(7.5-16.0)	8.0(5.3-10.6)	1.208	0.227
Blood loss (mL)	1500.0(1000.0-3000.0)	1350.0(1000.0-1700.0)	0.490	0.624
Warm ischemia time (min)	3.4(3.0-4.5)	3.5(3.0-5.0)	0.207	0.802
Cold ischemia time (min)	52.5(41.0-67.0)	55.5(35.0-69.0)	0.629	0.512
ALT(IU/L)	49.0(23.0-102.0)	40.0(26.0-80.8)	0.390	0.969
AST(IU/L)	80.0(48.0-133.0)	77.5(38.0-103.5)	1.216	0.224
GGT(IU/L)	58.0(29.0-109.0)	51.5(28.3-90.5)	0.510	0.610
ALP(IU/L)	132.0(106.0-205.0)	131.5(102.8-155.8)	0.497	0.619
TB(μmol/L)	263.0(156.0-336.0)	285.0(255.5-453.3)	1.556	0.120
Bile acids (μmol/L)	232.3(133.7-292.5)	188.4(117.9-371.0)	0.327	0.744
Creatinine (μmol/L)	69.0(57.0-132.0)	72.5(48.0-129.8)	0.288	0.774
White blood cell count (WBC, ×10^9/L)	5.6(3.8-8.7)	3.7(2.9-6.7)	1.543	0.123
Neutrophil percentage (N%)	77.4(66.2-86.7)	76.2(68.7-83.0)	0.680	0.497
Neutrophil count (×10^9/L)	4.0(2.6-7.2)	3.2(1.9-5.0)	1.387	0.166
CRP(ng/ml)	11.2(7.6-29.5)	12.3(6.0-22.6)	0.732	0.464
PCT(ng/ml)	0.6(0.3-1.3)	0.7(0.3-1.6)	0.314	0.754
IL-2(pg/ml)	1.0(0.7-1.6)	1.0(0.8-1.4)	0.086	0.931
IL-6(pg/ml)	19.8(8.2-38.1)	18.1(9.2-36.6)	0.052	0.959
IL-10(pg/ml)	6.2(2.5-14.3)	5.1(3.4-12.9)	0.173	0.863
TNF-α(pg/ml)	2.2(1.2-2.5)	1.9(1.4-2.9)	0.190	0.849
IFN-γ(pg/ml)	4.1(2.4-7.7)	3.5(2.3-8.5)	0.541	0.451

BMI, body mass index; MELD, model for end-stage liver disease; ALT, alanine aminotransferase; AST, aspartate aminotransferase; GGT, gamma-glutamyl transpeptidase; ALP, alkaline phosphatase; TB, total bilirubin; WBC, white blood cell; N%, neutrophilic granulocyte percentage; CRP, C-reactive protein; PCT, procalcitonin; IL, interleukin; TNF-α, tumor necrosis factor-α; IFN-γ, interferon-γ.

### Analysis of postoperative infections during the first month after LT

Within 1 month after LT, bacterial infection occurred in 26 of the 60 patients. The infection rate was significantly lower in the probiotic group than in the non-probiotic group (30.0% vs. 56.7%, respectively; P = 0.037). Although the frequencies of respiratory, intra-abdominal, biliary, wound, bloodstream, and urinary tract infections were all numerically lower in the probiotic group, only the difference in intra-abdominal infection reached statistical significance (6.7% vs. 33.3%; P = 0.024) (**Table 2**). Microbiological analysis identified 80 non-duplicate pathogenic strains during the first postoperative month, including 21 isolates in the probiotic group and 59 in the non-probiotic group. Gram-negative bacteria accounted for the majority of isolates (n = 50), whereas Gram-positive bacteria accounted for 30 isolates. The predominant Gram-negative organisms were *Klebsiella pneumoniae, Acinetobacter baumannii*, and *Pseudomonas aeruginosa*, while the most common Gram-positive organisms were *Enterococcus faecium, Staphylococcus epidermidis*, and *Staphylococcus aureus*. Most isolates were obtained from respiratory specimens, followed by abdominal drainage fluid and peripheral blood (**Supplementary Table 2**).

**Table 2:** Infection rates and distribution of infectious episodes in the probiotic and non-probiotic groups after liver transplantation.

Type of infection	Non-probiotic groups (n=30)	Probiotic groups (n=30)	χ2	P-value
Overall infection	17(56.7%)	9(30.0%)	4.344	0.037
Respiratory tract infection	11(36.7%)	5(16.7%)	3.068	0.080
Intra-abdominal infection	10(33.3%)	2(6.7%)	5.104	0.024
Biliary tract infection	3(10.0%)	1(3.3%)	0.268	0.605
Wound infection	2(6.7%)	1(3.3%)	0.001	1.000
Bloodstream infection	6(20.0%)	3(10.0%)	0.523	0.470
Urinary tract infection	2(6.7%)	1(3.3%)	0.001	1.000

### Perioperative inflammatory marker changes following LT

**Figure 1:**
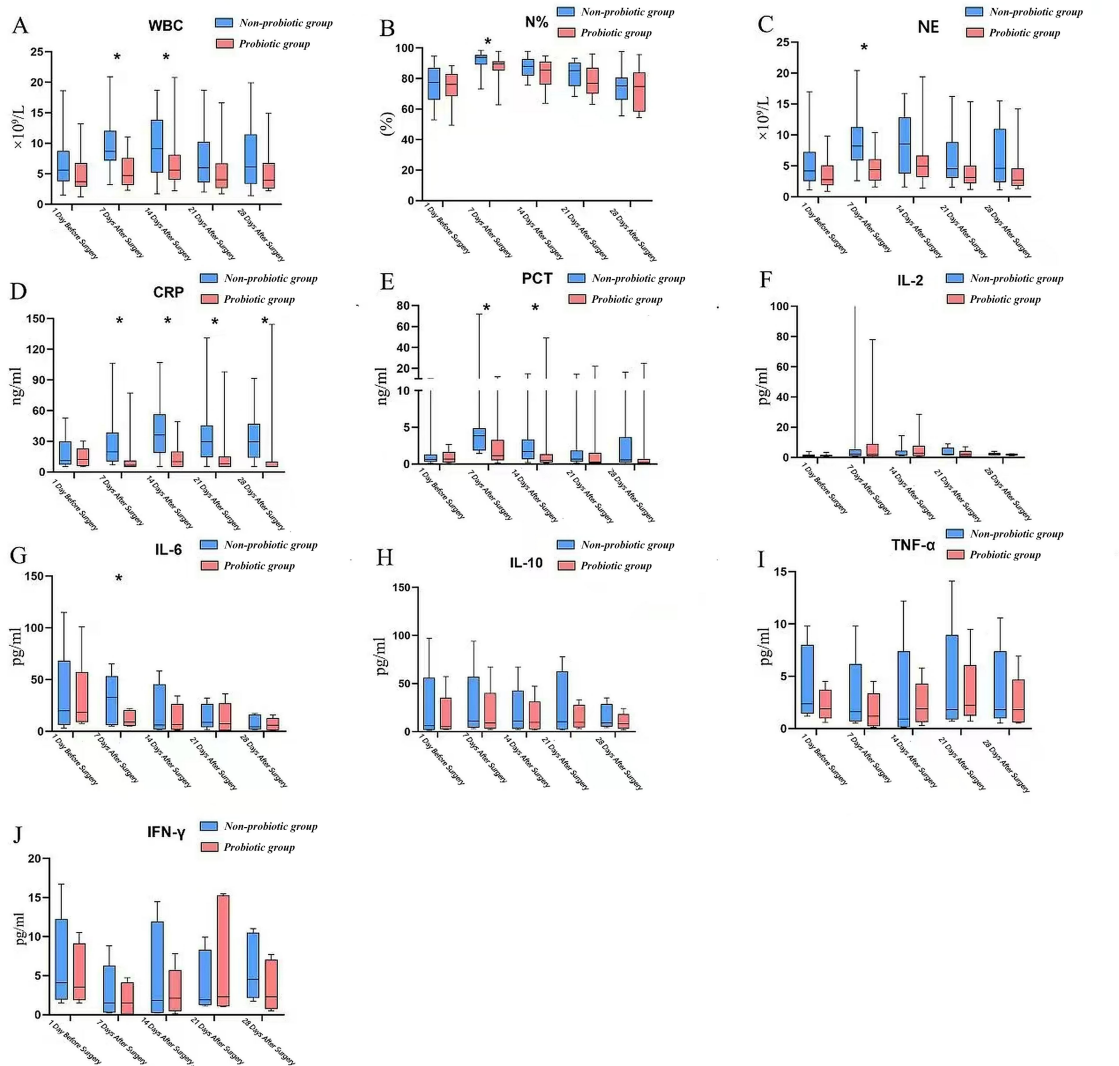
Perioperative trends of inflammatory markers in the two patient groups. **(A)** WBC trend; **(B)** N% trend; **(C)** neutrophil trend; **(D)** CRP trend; **(E)** PCT trend; **(F)** IL-2 trend; **(G)** IL-6 trend; **(H)** IL-10 trend; **(I)** TNF-α trend; **(J)** IFN-γ trend. P < 0.05. WBC, white blood cell; N%, neutrophil percentage; CRP, C-reactive protein; PCT, procalcitonin; IL-2, interleukin-2; IL-6, interleukin-6; IL-10, interleukin-10; TNF-α, tumor necrosis factor-α; IFN-γ, interferon-γ. * indicates a P value < 0.05.

Compared with the non-probiotic group, patients receiving probiotic intervention exhibited significantly lower levels of white blood cell count (WBC) on postoperative days (POD) 7 and 14, as well as lower neutrophil percentage and absolute neutrophil count on POD7, indicating a more rapid attenuation of the early systemic inflammatory response. In addition, C-Reactive Protein (CRP) levels remained consistently lower in the probiotic group at multiple postoperative time points, while procalcitonin (PCT) was significantly reduced on POD7 and POD14, further supporting a lower postoperative infectious burden. Among the measured cytokines, IL-6 was significantly lower in the probiotic group on POD7, whereas IL-2, IL-10, TNF-α, and IFN-γ did not show significant intergroup differences (**Figure 1**).

### One day before LT

**Figure 2:**
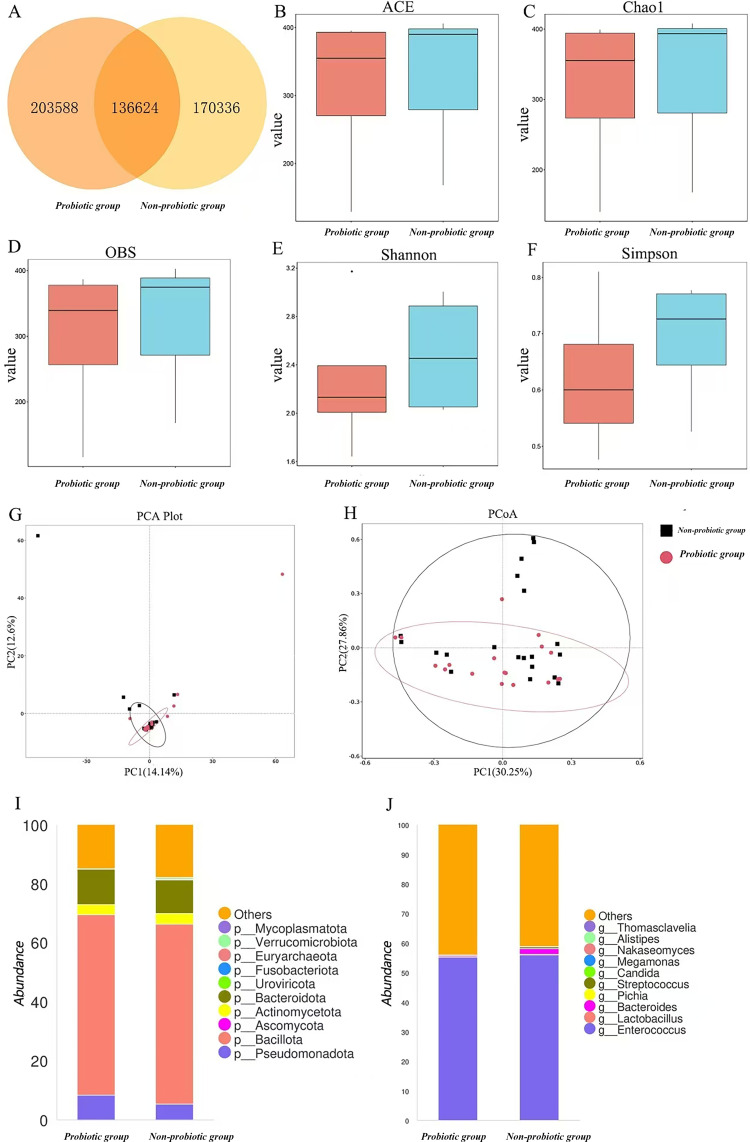
Analysis and comparison of fecal gut microbiota between the non-intervention and intervention groups one day before surgery. **(A)** Venn diagram showing the number of microbial genes in the two groups; **(B–F)** α-diversity analysis between the two groups using the ACE, Chao1, OBS, Shannon, and Simpson indices; **(G, H)** β-diversity analysis between the two groups based on PCA and PCoA; **(I)** composition and relative abundance of intestinal microbiota at the phylum level in the two groups; **(J)** composition and relative abundance of intestinal microbiota at the genus level in the two groups. *P < 0.05, **P < 0.01.

The Venn diagram showed that the non-probiotic group contained 170,336 unique genes, whereas the probiotic group harbored 203,588 unique genes, with 136,624 genes shared between both groups (**Figure 2A**). Alpha diversity analysis, including ACE, Chao1, Observed Species (OBS), Shannon, and Simpson indices, revealed no significant differences in species richness or evenness between the two groups (P > 0.05; **Figures 2B–F**). Similarly, beta diversity analysis using PCA and PCoA demonstrated partial overlap of the groups, indicating that the overall community structures were not completely distinct (**Figures 2G, H**). These results suggest that both alpha and beta diversity of the gut microbiota were comparable between the non-probiotic and probiotic groups prior to LT (P > 0.05). At the phylum level, the most abundant taxa in the non-probiotic group were *Firmicutes* (60.92%), *Bacteroidetes* (11.39%), *Proteobacteria* (5.44%), and *Actinobacteria* (3.58%), while in the probiotic group, the top four phyla were *Firmicutes* (61.20%), *Bacteroidetes* (11.95%), *Proteobacteria* (8.33%), and *Actinobacteria* (3.44%). At the genus level, *Enterococcus* (55.55%), *Bacteroides* (2.02%), *Klebsiella* (0.42%), and *Streptococcus* (0.15%) predominated in the non-probiotic group, whereas the probiotic group was dominated by *Enterococcus* (52.47%), *Bacteroides* (0.84%), *Klebsiella* (0.20%), and *Streptococcus* (0.08%) (**Figures 2I, J**). Overall, the gut microbiota composition was highly similar between the two groups at both the phylum and genus levels, with *Firmicutes* and *Enterococcus* predominating, respectively.

### Seven days after LT

**Figure 3:**
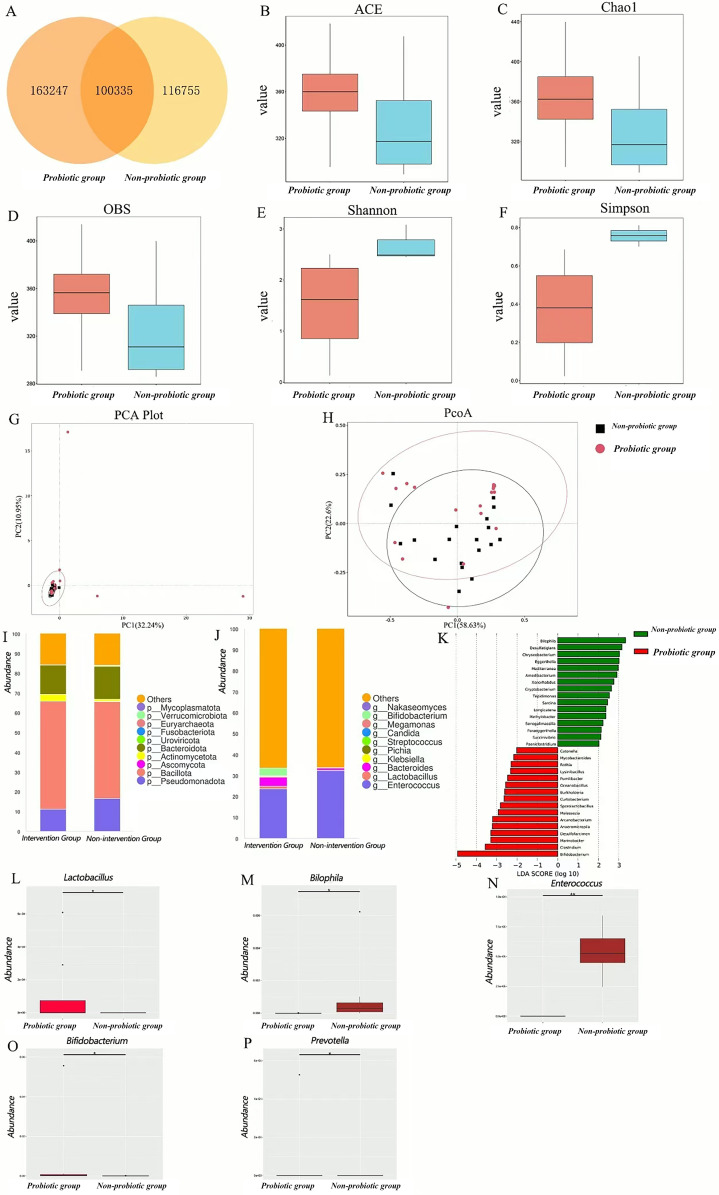
Analysis and comparison of fecal gut microbiota between the non-intervention and intervention groups seven days after surgery. **(A)** Venn diagram showing the number of microbial genes in the two groups; **(B–F)** α-diversity analysis between the two groups using the ACE, Chao1, OBS, Shannon, and Simpson indices; **(G, H)** β-diversity analysis between the two groups based on PCA and PCoA; **(I)** composition and relative abundance of intestinal microbiota at the phylum level in the two groups; **(J)** composition and relative abundance of intestinal microbiota at the genus level in the two groups; **(K)** LEfSe analysis, with the histogram showing taxa with LDA scores greater than the default threshold of 2, where green indicates the non-intervention group and red indicates the intervention group; **(L–P)** metastat analysis showing the top five taxa with significant differences between the two groups: **(L)** Lactobacillus, **(M)** Bilophila, **(N)** Enterococcus, **(O)** Bifidobacterium, and **(P)** Prevotella. *P < 0.05, **P < 0.01.

The Venn diagram showed that the non-probiotic group harbored 116,755 unique genes, and the probiotic group contained 163,247 unique genes, with 100,335 genes shared between them (**Figure 3A**). Total gut microbial gene counts decreased in both groups compared with preoperative levels. At the phylum level, *Firmicutes* remained dominant, although its relative abundance declined postoperatively. At the genus level, *Enterococcus* decreased in both groups, more markedly in the probiotic group (23.69% vs. 32.34%), while *Bifidobacterium* and *Lactobacillus* increased in the probiotic group compared with baseline. Alpha diversity metrics (ACE, Chao1, OBS, Shannon, Simpson) showed no significant differences between groups (P > 0.05; **Figures 3B–F**), and beta diversity analysis (PCA, PCoA) indicated partial overlap, suggesting overall community structures were comparable (**Figures 3G, H**). At the phylum level, the top four taxa in the non-probiotic group were *Firmicutes* (49.11%), *Proteobacteria* (16.61%), *Bacteroidetes* (16.57%), and *Actinobacteria* (0.99%), with *Enterococcus* (32.34%), *Bacteroides* (1.05%), *Lactobacillus* (0.16%), and *Klebsiella* (0.10%) predominating at the genus level. In the probiotic group, Firmicutes (54.71%), Bacteroidetes (14.59%), Proteobacteria (11.18%), and Actinobacteria (3.54%) were most abundant, with *Enterococcus* (23.69%), *Bacteroides* (4.38%), *Bifidobacterium* (3.62%), and *Lactobacillus* (1.16%) dominating (**Figures 3I, J**). LEfSe and MetaStat analyses identified taxa differing significantly between groups. LEfSe showed 16 genera enriched in each group, including *Clostridium*, *Klebsiella*, and *Enterobacter* in the non-probiotic group, and *Bifidobacterium*, *Clostridium*, and *Bacillus subtilis* in the probiotic group (**Figure 3K**). MetaStat confirmed enrichment of *Clostridium* and *Enterococcus* in the non-probiotic group, and *Lactobacillus*, *Bifidobacterium*, and *Prevotella* in the probiotic group (**Figures 3L–P**; P < 0.05).

### Fourteen days after LT

**Figure 4:**
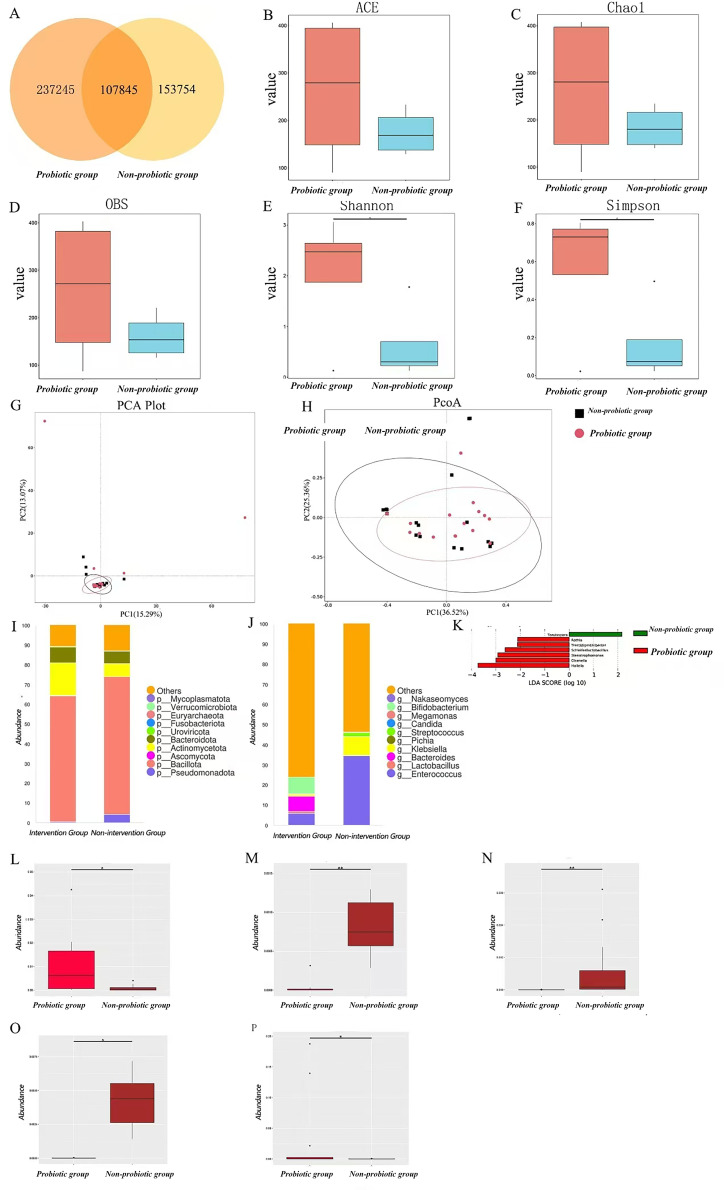
Analysis and comparison of fecal gut microbiota between the non-intervention and intervention groups fourteen days after surgery. **(A)** Venn diagram showing the number of microbial genes in the two groups; **(B–F)** α-diversity analysis between the two groups using the ACE, Chao1, OBS, Shannon, and Simpson indices; **(G, H)** β-diversity analysis between the two groups based on PCA and PCoA; **(I)** composition and relative abundance of intestinal microbiota at the phylum level in the two groups; **(J)** composition and relative abundance of intestinal microbiota at the genus level in the two groups; **(K)** LEfSe analysis, with the histogram showing taxa with LDA scores greater than the default threshold of 2, where red indicates the intervention group and blue indicates the non-intervention group; **(L–P)** metastat analysis showing the top five taxa with significant differences between the two groups: **(L)** Bifidobacterium, **(M)** Enterococcus, **(N)** Bilophila, **(O)** Klebsiella, and **(P)** Lactobacillus. *P < 0.05, **P < 0.01.

The Venn diagram showed that 153,754 genes were unique to the non-probiotic group and 237,242 to the probiotic group, with 107,845 genes shared between the two groups (**Figure 4A**). Compared with postoperative day 7, microbial gene numbers increased in both groups by day 14, with a faster recovery in the probiotic group. Alpha diversity analysis showed that the Shannon and Simpson indices were significantly higher in the probiotic group than in the non-probiotic group (*P* < 0.05), whereas the ACE, Chao1, and OBS indices did not differ significantly (*P* > 0.05) (**Figures 4B–F**), indicating greater microbial evenness in the probiotic group. Beta diversity analysis showed partial overlap between the two groups, suggesting that their overall microbial community structures were not completely distinct (**Figures 4G, H**). At postoperative day 14, *Firmicutes* remained the dominant phylum in both groups. In the non-probiotic group, the most abundant phyla were *Firmicutes* (69.68%), *Proteobacteria* (14.18%), *Actinobacteria* (6.67%), and *Bacteroidetes* (6.22%), while the dominant genera were *Enterococcus* (34.28%), *Klebsiella* (9.31%), *Streptococcus* (2.08%), and *Bacteroides* (0.25%). In the probiotic group, the leading phyla were *Firmicutes* (63.77%), *Actinobacteria* (16.48%), *Bacteroidetes* (7.81%), and *Proteobacteria* (0.49%), and the dominant genera were *Bifidobacterium* (8.21%), *Bacteroides* (7.39%), *Enterococcus* (5.77%), and *Lactobacillus* (1.16%) (**Figures 4I, J**). Overall, the non-probiotic group remained characterized by *Enterococcus* predominance and increased *Klebsiella* abundance, whereas the probiotic group showed enrichment of *Bifidobacterium* and reduced *Enterococcus* abundance. LEfSe and MetaStat analyses further identified significant intergroup differences. LEfSe detected one discriminative biomarker in the non-probiotic group, namely *Saccharomyces* spp., while six biomarker genera were enriched in the probiotic group, including *Lactobacillus*, *Howardella*, *Olsenella*, *Stenotrophomonas*, *Tractidigestivibacter*, and *Roseburia* (**Figure 4K**). MetaStat analysis showed enrichment of bile-tolerant bacteria, *Enterococcus*, and *Klebsiella* in the non-probiotic group, whereas *Lactobacillus* and *Bifidobacterium* were enriched in the probiotic group (**Figures 4L–P**; *P* < 0.05).

### Twenty-one days after LT

**Figure 5:**
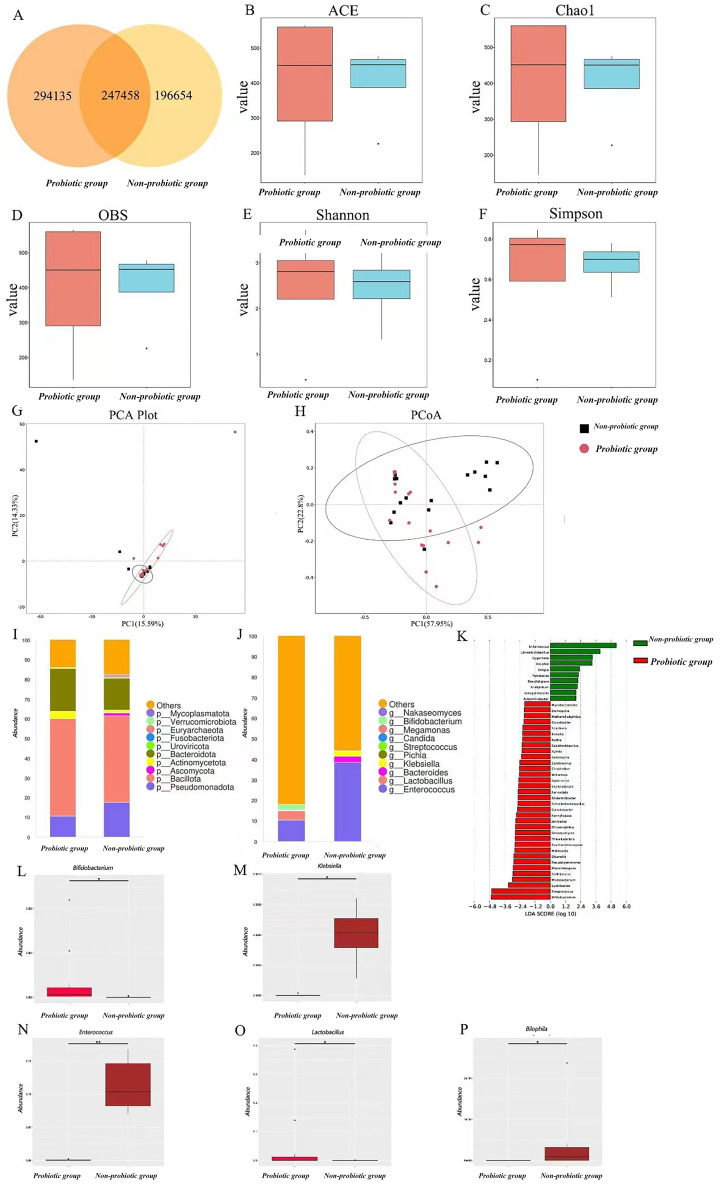
Analysis and comparison of fecal gut microbiota between the non-intervention and intervention groups twenty-one days after surgery. **(A)** Venn diagram showing the number of microbial genes in the two groups; **(B–F)** α-diversity analysis between the two groups using the ACE, Chao1, OBS, Shannon, and Simpson indices; **(G, H)** β-diversity analysis between the two groups based on PCA and PCoA; **(I)** composition and relative abundance of intestinal microbiota at the phylum level in the two groups; **(J)** composition and relative abundance of intestinal microbiota at the genus level in the two groups; **(K)** LEfSe analysis, with the histogram showing taxa with LDA scores greater than the default threshold of 2, where red indicates the intervention group and blue indicates the non-intervention group; **(L–P)** metastat analysis showing the top five taxa with significant differences between the two groups: **(L)** Bifidobacterium, **(M)** Klebsiella, **(N)** Enterococcus, **(O)** Lactobacillus, and **(P)** Bilophila. *P < 0.05, **P < 0.01.

The Venn diagram showed that 196,654 genes were unique to the non-probiotic group and 294,135 to the probiotic group, with 247,458 genes shared between the two groups (**Figure 5A**). By postoperative day 21, the total microbial gene count in both groups had exceeded the preoperative level. Alpha diversity analysis showed that ACE, Chao1, OBS, Shannon, and Simpson indices were all higher than baseline in both groups, indicating increased microbial richness and evenness after transplantation. However, none of these indices differed significantly between groups (*P* > 0.05) (**Figures 5B–F**). Beta diversity analysis likewise showed partial overlap between the two groups, suggesting that their overall microbial community structures were not completely distinct (**Figures 5G, H**). Firmicutes remained the predominant phylum in both groups. In the non-probiotic group, the major phyla were *Firmicutes* (43.88%), *Proteobacteria* (17.56%), *Bacteroidetes* (16.15%), and *Actinobacteria* (1.55%), while the dominant genera were *Enterococcus* (38.44%), *Bacteroides* (2.81%), *Klebsiella* (2.59%), and *Streptococcus* (0.05%). In the probiotic group, the leading phyla were *Firmicutes* (49.40%), *Bacteroidetes* (21.81%), *Proteobacteria* (10.63%), and *Actinobacteria* (3.75%), whereas the dominant genera were *Enterococcus* (10.34%), *Lactobacillus* (4.62%), *Bifidobacterium* (2.53%), and *Bacteroides* (0.19%) (**Figures 5I, J**). Overall, *Enterococcus* remained dominant in both groups but was far more abundant in the non-probiotic group. LEfSe and MetaStat analyses further identified significant intergroup differences. LEfSe revealed 10 biomarker taxa enriched in the non-probiotic group, including *Enterococcus*, bile-tolerant bacteria, and *Exiguobacterium*, whereas 34 taxa were enriched in the probiotic group, including *Bifidobacterium*, *Leptotrichia*, and *Pseudolysinimonas* (**Figure 5K**). MetaStat analysis similarly showed enrichment of *Klebsiella*, *Enterococcus*, and bile-tolerant bacteria in the non-probiotic group, while *Bifidobacterium* and *Lactobacillus* were enriched in the probiotic group (**Figures 5L–P**; *P* < 0.05).

### Twenty-eight days after LT

**Figure 6:**
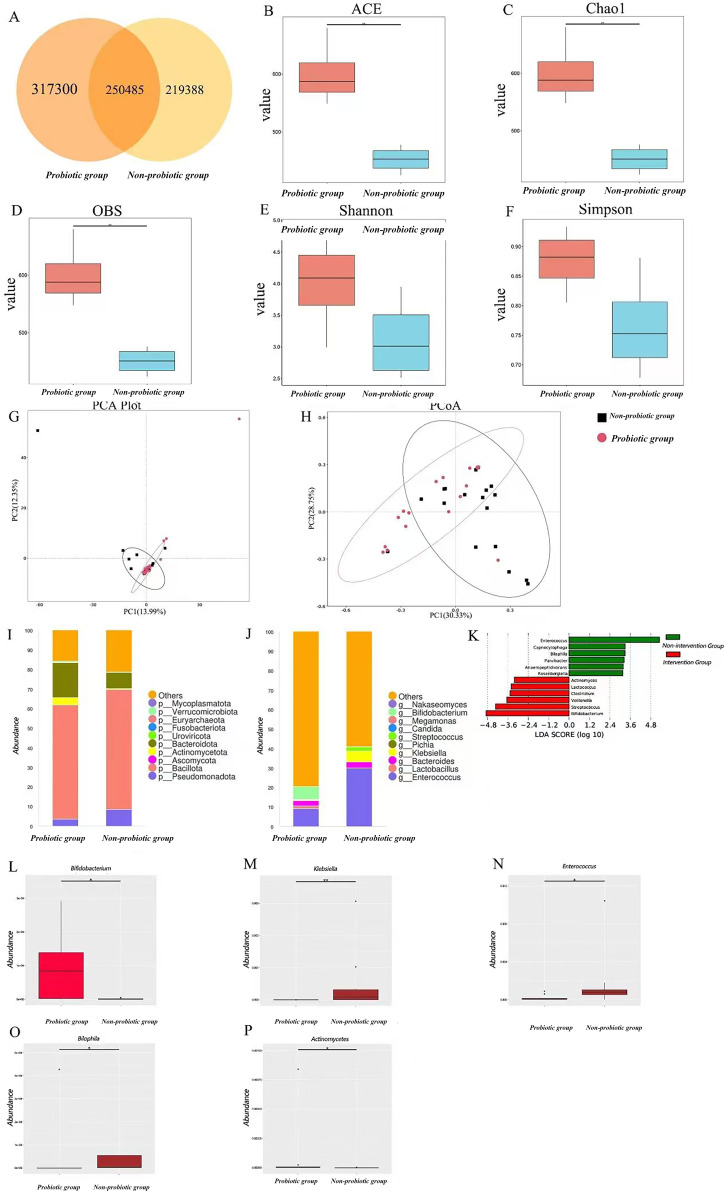
Analysis and comparison of fecal gut microbiota between the non-intervention and intervention groups twenty-eight days after surgery. **(A)** Venn diagram showing the number of microbial genes in the two groups; **(B–F)** α-diversity analysis between the two groups using the ACE, Chao1, OBS, Shannon, and Simpson indices; **(G, H)** β-diversity analysis between the two groups based on PCA and PCoA; **(I)** composition and relative abundance of intestinal microbiota at the phylum level in the two groups; **(J)** composition and relative abundance of intestinal microbiota at the genus level in the two groups; **(K)** LEfSe analysis, with the histogram showing taxa with LDA scores greater than the default threshold of 2, where red indicates the intervention group and blue indicates the non-intervention group; **(L–P)** metastat analysis showing the top five taxa with significant differences between the two groups: **(L)** Bifidobacterium, **(M)** Klebsiella, **(N)** Enterococcus, **(O)** Bilophila, and **(P)** Actinomyces. *P < 0.05, **P < 0.01.

The Venn diagram showed that 219,388 genes were unique to the non-probiotic group and 317,300 to the probiotic group, with 250,485 genes shared between the two groups (**Figure 6A**). Compared with postoperative day 21, the total number of gut microbial genes further increased in both groups by day 28. Alpha diversity analysis demonstrated that the ACE, Chao1, and Observed Species (OBS) indices were significantly higher in the probiotic group than in the non-probiotic group (*P* < 0.05), whereas the Shannon and Simpson indices did not differ significantly between groups (*P* > 0.05) (**Figures 6B–F**). These findings suggest that microbial richness was greater in the probiotic group, while community evenness was comparable between the two groups. Beta diversity analysis showed partial overlap between groups, indicating that the overall microbial community structures were not completely separated (**Figures 6G, H**). At the phylum level, Firmicutes remained the dominant phylum in both groups. In the non-probiotic group, the four most abundant phyla were Firmicutes (61.33%), Bacteroidetes (8.43%), Proteobacteria (8.42%), and Actinobacteria (0.34%). At the genus level, the dominant taxa were *Enterococcus* (30.00%), *Klebsiella* (5.53%), *Bacteroides* (3.16%), and *Streptococcus* (2.09%). In the probiotic group, the leading phyla were Firmicutes (58.28%), Bacteroidetes (17.76%), Actinobacteria (3.71%), and Proteobacteria (3.57%), while the predominant genera were *Enterococcus* (9.02%), *Bifidobacterium* (6.67%), *Bacteroides* (2.79%), and *Lactobacillus* (1.54%) (**Figures 6I, J**). Overall, although both groups remained dominated by Firmicutes at the phylum level and *Enterococcus* at the genus level, the abundance of *Enterococcus* was markedly higher in the non-probiotic group than in the probiotic group (30.00% vs. 9.02%). To further identify taxa that differed significantly between groups, LEfSe and MetaStat analyses were performed. LEfSe analysis identified six biomarker genera enriched in the non-probiotic group, including *Enterococcus*, bile-tolerant bacteria, and *Exiguobacterium*, whereas six biomarker genera were enriched in the probiotic group, including *Bifidobacterium*, *Lactobacillus*, and *Actinomyces* (**Figure 6K**). MetaStat analysis further showed that *Enterococcus*, bile-tolerant bacteria, and *Klebsiella* were enriched in the non-probiotic group, whereas *Actinomyces* and *Bifidobacterium* were enriched in the probiotic group (**Figures 6L–P**). These intergroup differences were statistically significant (*P* < 0.05).

### Analysis of antibiotic resistance genes in the two groups after LT

**Figure 7:**
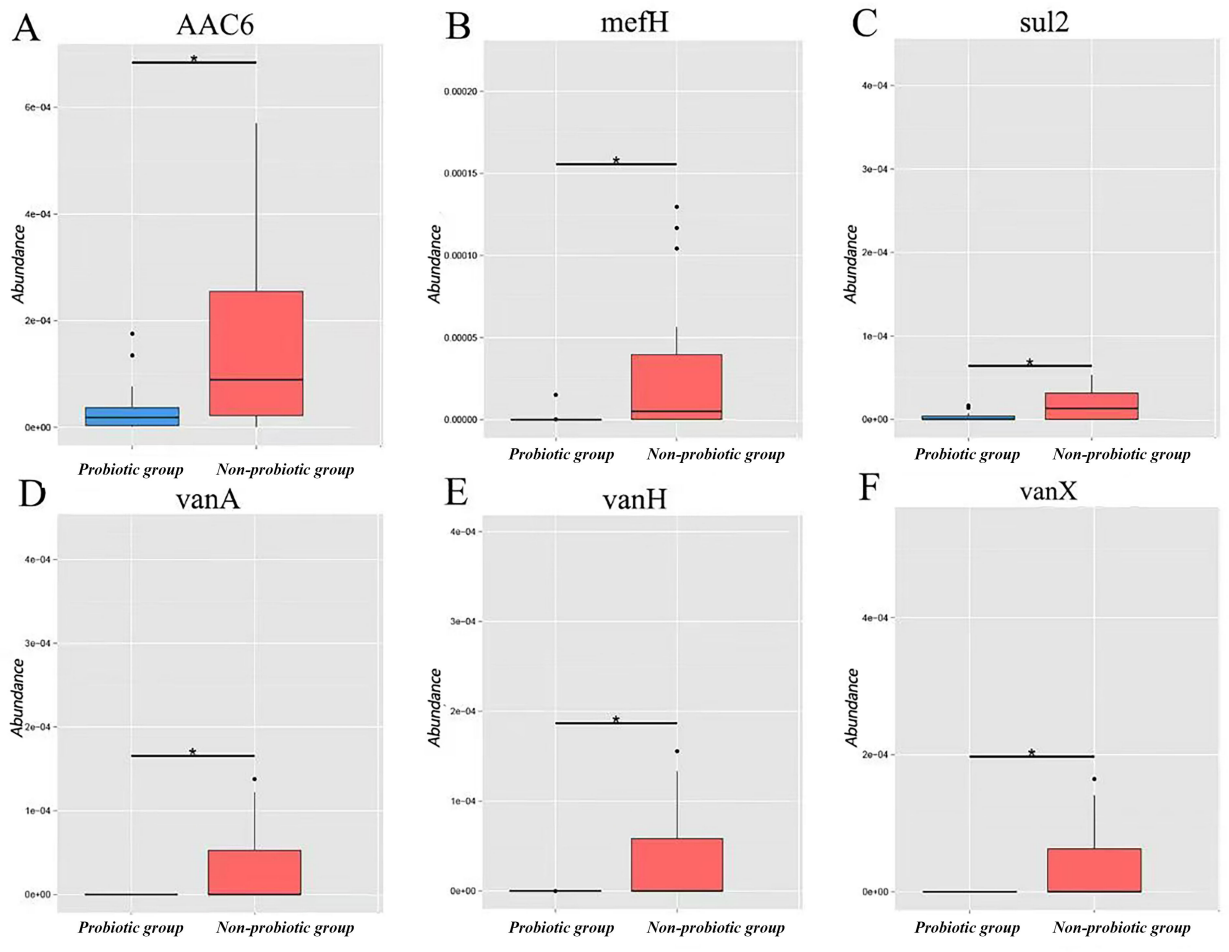
Metastat analysis of antibiotic resistance genes in the two groups after surgery. **(A)** aminoglycoside resistance gene AAC6; **(B)** macrolide resistance gene mefH; **(C)** sulfonamide resistance gene sul2; **(D)** glycopeptide resistance gene vanA; **(E)** glycopeptide resistance gene vanH; **(F)** glycopeptide resistance gene vanX. *P < 0.05.

To further characterize differences in the postoperative gut resistome between the two groups, we performed resistance gene profiling based on the Comprehensive Antibiotic Resistance Database (CARD). Compared with the probiotic group, the non-probiotic group showed significant enrichment of the aminoglycoside resistance gene AAC6, the macrolide resistance gene mefH, the sulfonamide resistance gene sul2, and the glycopeptide resistance genes vanA, vanH, and vanX (*P* < 0.05) (**Figures 7A–F**).

## Discussion

Advances in LT have substantially improved postoperative outcomes, with reported one-year survival rates approaching 90%–95% (Liver, 2024; Kwong et al., 2025). Nevertheless, patients with end-stage liver disease complicated by hepatic encephalopathy, hepatorenal syndrome, or severe systemic infections continue to experience markedly lower survival (Arvaniti et al., 2010). Evidence from both domestic and international cohorts indicates that critically ill recipients with significant comorbidities achieve post-transplant survival rates of only 60%–80% (Duan et al., 2013; Moon et al., 2017; Li et al., 2023). Among the factors affecting prognosis, postoperative complications—particularly infectious events—remain a leading cause of mortality and a critical determinant of long-term patient quality of life (Lim et al., 2018; Zhang et al., 2021). Specifically, the presence of perioperative abdominal or pulmonary infections has been associated with postoperative mortality exceeding 40%, compared with approximately 20% in patients without such infections (Zhang et al., 2021).

The human gastrointestinal tract harbors a complex microbial ecosystem that coexists symbiotically with the intestinal mucosa, contributing critically to immune regulation, metabolic homeostasis, and gastrointestinal barrier function in healthy individuals (Jandhyala et al., 2015; Chen et al., 2023). Dysbiosis of the gut microbiota has been increasingly recognized as a key factor that heightens susceptibility to infections (Ancona et al., 2021; Velikova et al., 2026). Alterations in microbial composition and abundance can promote bacterial translocation and the overgrowth of opportunistic pathogens, particularly Gram-negative bacteria, while increasing intestinal permeability (Di Vincenzo et al., 2024). This may activate innate immune signaling pathways, including Toll-like receptors and NOD-like receptors, leading to NF-κB-mediated production of proinflammatory cytokines and chemokines, which enter the portal circulation and exacerbate hepatic inflammation and disease progression (Carotti et al., 2015; Tripathi et al., 2018). Patients with liver failure often present with severe systemic illness, and postoperative gut microbial imbalances are typically more pronounced (Song et al., 2023). Microbiota-targeted interventions, such as the administration of probiotics, have been shown to partially restore microbial equilibrium, representing a potential strategy to reduce postoperative infections following LT (Shimizu et al., 2021; Abenavoli et al., 2023). To our knowledge, the present study is the first to employ metagenomic sequencing to investigate the effects of microbiota-based interventions on early postoperative infection and recovery in patients undergoing LT for liver failure.

Following LT, the probiotic group showed a significantly lower overall incidence of postoperative infections, particularly intra-abdominal infections, than the non-probiotic group. During the first postoperative month, inflammatory markers recovered more rapidly in the probiotic group, as reflected by lower white blood cell count, neutrophil percentage, absolute neutrophil count, CRP, and procalcitonin levels, mainly on postoperative days 7 and 14. Notably, CRP levels remained consistently lower throughout the first month. These findings suggest that probiotic administration may facilitate early postoperative inflammatory recovery and reduce infection risk, especially intra-abdominal infections. However, the relationships among gut microbiota dysbiosis, antibiotic exposure, and postoperative infection are complex and likely bidirectional (Nichols et al., 2025). Delayed microbiota reconstruction may increase infection susceptibility by reducing colonization resistance, impairing intestinal barrier function, and promoting opportunistic pathogen expansion (Kim et al., 2017; Duan et al., 2022). Conversely, postoperative infections often require intensive or prolonged antibiotic therapy, which may further reshape the gut microbiota and select for antibiotic-resistant organisms (Pilmis et al., 2020). Therefore, the altered microbial profiles and enriched antibiotic resistance genes observed in this study may reflect both microbiota-associated vulnerability to infection and antibiotic-driven selection after infection treatment. Given the retrospective observational design, causality cannot be inferred. Prospective studies with standardized antibiotic protocols, detailed exposure data, and time-dependent analyses are warranted to clarify these causal relationships after LT. In addition, Liver failure subtype may also affect gut microbiota reconstruction after LT. Owing to the limited sample size, subtype-stratified sensitivity analysis was not feasible. Larger prospective studies are needed to assess whether microbiota recovery and probiotic-related clinical benefits differ across liver failure subtypes.

Dysbiosis of the gut microbiota is typically characterized by reduced microbial diversity, decreased overall abundance, and overrepresentation of potential pathogens. In our study, the non-probiotic group exhibited notable overgrowth of opportunistic bacteria, including Bilophila and Enterococcus species. *Bilophila*, an opportunistic pathogen, can exacerbate intestinal inflammation and, in synergy with high-fat diets, has been linked to impaired gut barrier function and altered bile acid metabolism (Natividad et al., 2018). Post-LT infections were predominantly associated with *Staphylococcus aureus*, *Enterococcus faecium*, *Escherichia coli*, and *Pseudomonas aeruginosa (*Chea et al., 2021; Guo et al., 2025*).* Surgical site infections were mainly caused by *Pseudomonas aeruginosa, Staphylococcus aureus*, and *Enterococcus species (*Chea et al., 2021*)*. Longitudinal analysis of gut microbiota demonstrated that α-diversity at postoperative days 21 and 28 increased relative to preoperative levels in both groups, with the probiotic group showing a greater magnitude of recovery. From an ecological perspective, higher microbial diversity is closely linked to ecosystem stability and functional resilience, suggesting that LT partially restores preoperative gut dysbiosis and impaired ecological function in patients with liver failure (Lozupone et al., 2012; Fassarella et al., 2021). The administration of probiotics further enhanced this recovery in the probiotic group. The lower incidence of postoperative infections in the probiotic group likely reduced the need for antibiotics, which may explain the observed differences in α-diversity between groups. In contrast, β-diversity analyses did not reveal significant differences between groups at any postoperative time point, which may be attributable to the limited sample size. Overall, gut microbial diversity gradually recovered after LT in both groups, with the probiotic group exhibiting a faster and more pronounced restoration.

Liver failure subtype may also influence gut microbiota reconstruction after liver transplantation. Patients with acute liver failure, chronic liver failure, and acute-on-chronic liver failure may differ in disease duration, systemic inflammation, intestinal barrier dysfunction, nutritional status, bile acid metabolism, and previous antibiotic exposure, all of which can affect gut microbial composition and probiotic response. Owing to the limited sample size, we were unable to perform a robust subtype-stratified sensitivity analysis in the present study. Future prospective studies with larger cohorts should evaluate whether microbiota recovery patterns and the clinical benefits of probiotic supplementation differ among liver failure subtypes.

Antibiotics are a cornerstone for preventing and controlling postoperative infections after LT; however, their use can disrupt gut microbial homeostasis and promote the emergence of antimicrobial resistance (Taylor et al., 2025). In this study, we assessed antibiotic resistance genes (ARGs) in the gut microbiota of both groups using the Comprehensive Antibiotic Resistance Database (CARD). The non-probiotic group showed higher abundances of ARGs, including AAC6, mefH, sul2, vanA, vanH, and vanX. In contrast, the reduced abundance of these resistance genes in the probiotic group may be associated with decreased enrichment of opportunistic pathogens, particularly *Enterococcus* and *Klebsiella*, following more favorable microbiota reconstruction. AAC6 encodes an aminoglycoside-modifying enzyme (Ramirez et al., 2013; Manoharan et al., 2022) reported that Enterococcus species can acquire high-level aminoglycoside resistance via this mechanism. mefH is an efflux pump protein conferring macrolide resistance, previously detected in Streptococcus mitis and Enterococcus species (Luna et al., 1999). vanA, a homolog of D-Ala-D-Ala ligase, synthesizes D-Ala-D-Lac, reducing vancomycin binding affinity. vanH, a D-specific α-keto acid dehydrogenase, generates D-lactate, and vanX, a D,D-dipeptidase, produces peptidoglycan substrates; collectively, VanA, VanH, and VanX mediate high-level vancomycin resistance in Enterococcus (Marshall et al., 1998). The enrichment of these genes in the non-probiotic group corresponds with the observed postoperative increase in Enterococcus abundance, suggesting that this genus is a key contributor to ARG accumulation. sul2, encoding a sulfonamide-resistant dihydropteroate synthase, is commonly found in Gram-negative bacteria such as *Escherichia coli*, *Klebsiella pneumoniae*, and *Acinetobacter baumannii (*de Los Santos et al., 2021; Bala et al., 2023; Guo et al., 2024*)*. The higher prevalence of these pathogens in the non-probiotic group post-transplantation aligns with the enrichment of sul2, indicating a potential link between ARGs and postoperative infection risk. Probiotic supplementation may mitigate this effect by promoting the growth of beneficial gut bacteria, suppressing opportunistic pathogens, and reducing bacterial translocation and infection incidence. Collectively, these findings provide mechanistic insight into how probiotic intervention could modulate gut resistome dynamics and help prevent postoperative infections in liver transplant recipients with liver failure.

This study has several limitations. First, this was a retrospective, single-center study with a relatively small sample size, which may have introduced selection bias, limited the generalizability of the findings, and reduced the statistical power to detect subtle microbiota-related differences. Second, the follow-up period was restricted to the first month after LT; therefore, the long-term effects of postoperative gut microbiota reconstruction and probiotic intervention could not be evaluated. Third, although we observed associations among gut microbial alterations, antibiotic resistance genes, and postoperative infections, the observational design precludes definitive causal inference. Finally, mechanistic validation was lacking, and further multicenter prospective studies with longer follow-up are needed to confirm these findings and clarify the underlying biological mechanisms.

## Conclusion

LT in patients with liver failure was accompanied by dynamic gut microbiota reconstruction during the first postoperative month. Although microbial diversity gradually recovered, patients without probiotic intervention showed persistent dysbiosis, with enrichment of opportunistic pathogens such as *Enterococcus* and *Klebsiella* and increased antibiotic resistance genes. In contrast, probiotic supplementation was associated with a more balanced microbial profile, increased abundance of beneficial bacteria such as *Bifidobacterium* and *Lactobacillus*, and fewer postoperative infections. These findings suggest that early gut microbiota alterations may be associated with infectious complications after LT. However, the underlying causal mechanisms remain unclear due to the lack of mechanistic validation. Future prospective studies incorporating *in vivo* or *in vitro* experiments are warranted.

## Data Availability

The original contributions presented in the study are included in the article/**Supplementary Material**. Further inquiries can be directed to the corresponding author.
