# Supplementary material for: Gut microbiota reconstruction after liver transplantation and its association with early postoperative infections in patients with liver failure
Source: Front Cell Infect Microbiol. 2026 Jun 8;16:1845273. doi: 10.3389/fcimb.2026.1845273 (PMC13284119; doi:10.3389/fcimb.2026.1845273)
Supplement: Supplementary file 1 [file Table1.docx]

Tabel S1. The diagnostic criteria of postoperative infections after LT

| **Postoperative infections after LT** | **Diagnostic criteria** |
| --- | --- |
| Respiratory tract infections | （1）Cough, sputum production, or worsening of pre-existing respiratory symptoms, with or without purulent sputum, dyspnea, chest pain, or hemoptysis;  （2）Fever;  （3）Signs of pulmonary consolidation or moist rales on auscultation;  （4）Peripheral WBC count >10 × 10^9/L or <4 × 10^9/L;  （5）Chest imaging showing new patchy infiltrates, interstitial changes, ground-glass opacities, or lobar or segmental consolidation, with or without pleural effusion.  Respiratory tract infection was diagnosed when any one of criteria (1) – (4) was present together with criterion (5), after exclusion of noninfectious diseases. |
| Intra-abdominal infections | Based on the patient’s chief complaints and history of present illness, the presence of fever, migratory right lower abdominal pain on physical examination, abdominal tenderness or rebound tenderness, increased abdominal muscle tension, and cessation of defecation and flatus were considered. Laboratory findings included elevated WBC count, N%, CRP, and PCT. Positive imaging findings included intra-abdominal abscess or fluid collection, and bacterial culture of abdominal drainage fluid was positive, with infections at other sites excluded. |
| Biliary tract infections | (A). Systemic inflammation:  (1) Fever (body temperature >38°C) and/or chills;  (2) Laboratory findings: WBC count <4 × 10^9/L or >10 × 10^9/L, and CRP ≥1 g/L.  (B). Cholestasis:  (1) Jaundice [total bilirubin (TB) ≥34.2 μmol/L];  (2) Laboratory findings: alkaline phosphatase (ALP) >1.5 × the upper limit of normal, gamma-glutamyl transpeptidase (GGT) >1.5 × the upper limit of normal, aspartate aminotransferase (AST) >1.5 × the upper limit of normal, and alanine aminotransferase (ALT) >1.5 × the upper limit of normal.  (C). Imaging findings:  (1) Biliary dilatation;  (2) Imaging evidence of the etiology (e.g., stricture, stones, tumor, stent, etc.).  (A) definite diagnosis can be established when one criterion from each of categories (A), (B), and (C) is met. |
| Surgical site infections | (1) Purulent drainage from the surgical incision, or pathogens cultured from fluid or tissue obtained from the incision, or the presence of symptoms or signs of infection, including local redness, swelling, heat, and pain;  (2) Etiological or pathological evidence obtained by puncture drainage, surgical exploration, or other procedures, or imaging findings indicating infection of the incisional tissue, organ, or cavity. |
| Urinary tract infections | (1) Manifestations such as urinary frequency, painful urination, urinary urgency, renal area pain, and fever;  (2) Urine culture: for clean-catch midstream urine culture, the bacterial colony count was >10^5 cfu/mL in women and >10^4 cfu/mL in men; for urine specimens collected by catheterization, the bacterial colony count was >10^4 cfu/mL in each patient;  (3) Functional or structural abnormalities of the genitourinary tract detected by imaging examinations such as urinary CT, ultrasonography, urography, or abdominal plain radiography, or the presence of other diseases associated with susceptibility to infection. |
| Bloodstream infections | Bacteria detected in the bloodstream, with a body temperature >38°C or <36°C, together with one of the following conditions: (1) the presence of an invasive portal of entry or a metastatic focus; (2) systemic toxic symptoms without an obvious site of infection; (3) rash or petechiae, hepatosplenomegaly, or increased peripheral neutrophils with a left shift that cannot be explained by other causes; (4) systolic blood pressure <90 mmHg or a decrease of >40 mmHg; or (5) isolation of pathogenic microorganisms from blood culture (if the organism is a skin colonizer, positive results from bilateral or multiple cultures are required), or detection of pathogenic microbial antigens, can be diagnosed as bloodstream infection. |

**Note:** Detection of bacterial infections after liver transplantation: Postoperatively, deep sputum (or bronchoalveolar lavage fluid), nasopharyngeal swabs, peripheral blood, bile, abdominal drainage fluid, wound drainage fluid, urine, and anal swabs were collected for bacterial culture. In patients with suspected intravenous catheter-related infection, the removed catheter tip was also cultured. If culture results were positive or infection was clinically suspected, cultures were repeated every 3 days until infection was controlled or cultures became negative.

LT liver transplantation; WBC white blood cell; N% neutrophil percentage; CRP C-reactive protein; PCT procalcitonin; TB total bilirubin; ALP alkaline phosphatase; GGT gamma-glutamyl transpeptidase; AST aspartate aminotransferase; ALT alanine aminotransferase. CT Computed Tomography.
