# Supplementary material for: Gut microbiota reconstruction after liver transplantation and its association with early postoperative infections in patients with liver failure
Source: Front Cell Infect Microbiol. 2026 Jun 8;16:1845273. doi: 10.3389/fcimb.2026.1845273 (PMC13284119; doi:10.3389/fcimb.2026.1845273)
Supplement: Supplementary file 2 [file Table2.docx]

**Table S2. Positive Bacterial Cultures and Distribution**

| Bacteria | N | Sputum / BALF / Throat swab | | Peritoneal drainage fluid | | Peripheral blood | | Bile | | Anal swab/ Fecal sample | | Urethra / Wound | |
| --- | --- | --- | --- | --- | --- | --- | --- | --- | --- | --- | --- | --- | --- |
|  |  | NP | P | NP | P | NP | P | NP | P | NP | P | NP | P |
| Gram-negative bacteria | 50 | 23 | 9 | 2 | 0 | 4 | 2 | 2 | 1 | 2 | 1 | 3 | 1 |
| *Klebsiella pneumoniae* | 19 | 9 | 2 | 2 | 0 | 1 | 0 | 2 | 0 | 2 | 0 | 1 | 0 |
| *Acinetobacter baumannii* | 12 | 7 | 3 | 0 | 0 | 1 | 0 | 0 | 0 | 0 | 0 | 0 | 1 |
| *Pseudomonas aeruginosa* | 6 | 1 | 1 | 0 | 0 | 0 | 1 | 0 | 1 | 0 | 0 | 2 | 0 |
| *Stenotrophomonas maltophilia* | 4 | 3 | 1 | 0 | 0 | 0 |  | 0 | 0 | 0 | 0 | 0 | 0 |
| *Escherichia coli* | 3 | 2 | 0 | 0 | 0 | 1 | 0 | 0 | 0 | 0 | 0 | 0 | 0 |
| *Enterobacter cloacae* | 4 | 1 | 1 | 0 | 0 | 0 | 1 | 0 | 0 | 0 | 1 | 0 | 0 |
| *Burkholderia* | 1 | 1 | 0 | 0 | 0 | 0 | 0 | 0 | 0 | 0 | 0 | 0 | 0 |
| *Klebsiella pneumoniae* | 1 | 0 | 0 | 0 | 0 | 1 | 0 | 0 | 0 | 0 | 0 | 0 | 0 |
| Gram-positive bacteria | 30 | 3 | 1 | 11 | 4 | 5 | 1 | 1 | 0 | 1 | 0 | 2 | 1 |
| *Enterococcus faecalis* | 15 | 1 | 0 | 9 | 2 | 0 | 0 | 1 | 0 | 0 | 0 | 2 | 0 |
| *Staphylococcus epidermidis* | 4 | 1 | 0 | 0 | 1 | 1 | 1 | 0 | 0 | 0 | 0 | 0 | 0 |
| *Staphylococcus aureus* | 3 | 1 | 0 | 0 | 0 | 1 | 0 | 0 | 0 | 0 | 0 | 0 | 1 |
| *hemolytic staphylococci* | 2 | 0 | 0 | 0 | 1 | 1 | 0 | 0 | 0 | 0 | 0 | 0 | 0 |
| *Staphylococcus aureus* | 2 | 0 | 0 | 1 | 0 | 1 | 0 | 0 | 0 | 0 | 0 | 0 | 0 |
| *Corynebacterium banding* | 2 | 1 | 0 | 1 | 0 | 0 | 0 | 0 | 0 | 0 | 0 | 0 | 0 |
| *Staphylococcus capitis* | 1 | 0 | 0 | 0 | 0 | 1 | 0 | 0 | 0 | 0 | 0 | 0 | 0 |
| *Clostridium difficile* | 1 | 0 | 0 | 0 | 0 | 0 | 0 | 0 | 0 | 1 | 0 | 0 | 0 |
| Total | 80 | 26 | 10 | 13 | 4 | 9 | 3 | 3 | 1 | 3 | 1 | 5 | 2 |

NP non-probiotic group；P probiotic group.
